# Impact of the Variable Killer Ig-Like Receptor–Human Leukocyte Antigen Interactions on Natural Killer Cell Cytotoxicity Toward Foreign CD4 T Cells

**DOI:** 10.3389/fimmu.2018.01588

**Published:** 2018-07-09

**Authors:** Jef Hens, Odin Goovaerts, Ann Ceulemans, Wim Jennes, Luc Kestens

**Affiliations:** ^1^Department of Biomedical Sciences, Institute of Tropical Medicine, Antwerp, Belgium; ^2^Department of Biomedical Sciences, University of Antwerp, Antwerp, Belgium

**Keywords:** allogeneic natural killer cell responses, killer Ig-like receptor–human leukocyte antigen ligand mismatch, *in vitro* natural killer-CD4 cocultures, human immunodeficiency virus-1 patients, HIV transmission

## Abstract

**Background:**

Natural killer (NK) cells are known to mount a response against foreign target cells, where the absence of the dominant inhibitory killer Ig-like receptor (KIR)–human leukocyte antigen (HLA) interaction immensely lowers the threshold for NK cell activation. NK cells could thus constitute a vital part in the mucosal defense against cell-associated sexually transmitted diseases. Here, we performed a detailed analysis of hitherto unexplored KIR–HLA-incompatible NK cell interactions.

**Methods and findings:**

*In vitro*, healthy NK cells were cocultured with CD4+ T cells derived from human immunodeficiency virus-1 patients, and the KIR-specific NK cell cytotoxicity was measured using flow cytometry. Genotyping of KIR and HLA predicted the KIR–HLA interactions occurring during these 124 allogeneic encounters. KIR2DL1+ NK cells were seen as the strongest intrinsic responders in the absence of their ligand with a 3.2-fold increase in KIR2DL1+ NK cells in the total NK cell response. An association between the size of the alloreactive NK cell population and the amount of CD4+ T cell death (*p* = 0.0023) and NK cell degranulation (*p* = 0.0036) was only present in NK cell donors with an activating KIR haplotype.

**Conclusion:**

We demonstrate differences in the activating effect of KIR–HLA incompatibility according to the KIR involved, with KIR2DL1 as the strongest responder. An activating KIR haplotype optimized the contribution of KIR–HLA-incompatible NK cells in the total NK cell response.

## Introduction

Natural killer (NK) cells provide a first line of defense against malignant or virally infected cells. This process is orchestrated by the receptor–ligand interactions upon encountering the target cell. The balance between inhibitory (1, 2) and activating (3–7) interactions will determine the cytotoxic behavior of the NK cell. NK cells mainly interact through receptors of the killer Ig-like receptor (KIR) family, which are categorized by their ability to transmit an inhibitory (iKIR) or an activating (aKIR) signal (1–8). On the side of the target cell, ligands for each iKIR are found on the human leukocyte antigen (HLA) class I molecules. Ligands for the KIR2DL1–3 receptors are situated on the HLA-C molecule, binding KIR2DL1 in the presence of a HLA-C2 motif, whereas a C1 motif will bind KIR2DL2 and -3 (9) KIR3DL1 binds HLA-A and -B molecules with a Bw4 motif (10). By contrast, ligands of activating receptors are generally restricted to tumor- or virally infected cells and are directly expressed on the membrane or as tumoral/viral peptides presented in the peptide-binding groove of HLA-molecules (11–15). Accordingly, NK cell activation is restricted to encounters with transformed or virally infected cells while tolerating healthy autologous cells.

The absence of the dominant HLA inhibitory signal on the target will bring the iKIR+ NK cell to the brink of activation, with only an activating signal needed to push it over the edge into a cytotoxic state. This principle has successfully been applied in stem cell transplantation for the treatment of leukemia by the use of haploidentical HLA donors. The constructed KIR–HLA mismatch resulted in “alloreactive” cytotoxic NK cell responses against the remaining leukemic cells while leaving the healthy recipient cells unharmed (16–18). As the activating “transformed” aspect thus allowed the selective killing of unwanted cells, it was the avoidance of the inhibitory “donor HLA” threshold that greatly amplified the response, resulting in improved clinical outcome. As a result of viral infection, cells likewise signal their deprived state by downregulating their HLA expression to accommodate anti-viral immunity. Nonetheless, viruses such as human immunodeficiency virus-1 (HIV) are able to preserve the expression of HLA-C and, to a lesser extent, HLA-B by viral proteins (19–21), hampering NK cell responsivity against these infected cells (22, 23).

During sexual HIV transmission, infected donor CD4+ T-cells are necessary to efficiently transfer the virus (24). As viral HLA modulation only influences the donor’s HLA repertoire, it renders itself vulnerable for recipient NK cell responses. In fact, resistance to HIV in serodiscordant couples has been related to a genetic KIR–HLA “mismatch” between the partners (25). This highlights the potential of a local (26) protective alloreactive NK cell response induced by a KIR–HLA mismatch. However, very little is known about how the different individual KIR–HLA interactions influence the NK cell response against allogeneic CD4+ T-cells (27). In this study, we examined the various allogeneic KIR–HLA interactions between NK cells derived from healthy individuals and CD4+ T-cells derived from HIV patients. Combining genetic and phenotypical data, we defined the strongest and weakest allogeneic KIR–HLA interactions, as well as their respective contribution to the total NK cell response.

## Materials and Methods

### Study Population

A total of 62 healthy individuals and 62 HIV patients were recruited as donors for the isolation of NK and CD4+ T-cells, respectively. NK cells were isolated from buffy coats obtained from healthy individuals at the Blood Transfusion Centre (Rode Kruis-Vlaanderen, Mechelen, Belgium). CD4+ T-cells were isolated from blood samples obtained from HIV patients at the Institute of Tropical Medicine (ITM). HIV patient selection criteria was a decent CD4+ T-cell count (≥500 cells/mm^3^). Approval for the use of HIV patients blood was granted by the ITM’s Institutional Review Board. General informed consent was obtained from all participating HIV patients (Policy number 99.002.067).

### *In Vitro* Cocultures

#### Experimental Design

Natural killer cells derived from healthy donors were cocultured with CD4+ T-cells derived from HIV patients. To maximize the amount of possible KIR–HLA combinations, NK cells were always cocultured in parallel with CD4+ T-cells from two different patients. In turn, the CD4+ T-cells of each HIV patient were paired with the NK cells of two healthy donors, resulting in four unique NK–CD4 encounters (Figure S1 in Supplementary Material). With 62 NK− and 62 CD4+ T-cell donors included in the study, this design resulted in a total of 124 NK–CD4 cocultures. All biosafety procedures to work in a BSL-2 were applied.

#### Isolation and Cultivation of NK Cells

Peripheral blood mononuclear cells (PBMCs) were isolated using a density gradient centrifugation (Ficoll-Paque PLUS, GE healthcare Life Sciences) from whole blood. The PBMCs underwent negative magnetic separation to isolate the NK cells (MidiMACS™ Separator, Miltenyi Biotec). NK cells were incubated in R10 [RPMI (LONZA) containing 100 U/ml penicillin, 100 µg/ml streptomycin, and 10% fetal bovine serum] and stimulated with 200 U/ml IL-2 (Gentaur) for 3 days at 37°C, 5% CO_2_.

#### Isolation and Cultivation of CD4+ T-Cells

Peripheral blood mononuclear cells were separated from HIV patient blood using a density gradient centrifugation (Ficoll-Paque) and were used to isolate CD4+ T-cells using a positive magnetic separation column (MiniMACS™ Separator, Miltenyi Biotec). The CD4+ T-cells were stimulated with 1 µg/ml phytohemagglutinin (PHA, Remel) and 100 U/ml IL-2 (Gentaur). All CD4+ T-cells were cultured overnight in R10.

#### Allogeneic *In Vitro* NK-CD4 Cocultures

After primary incubation, NK cells and CD4+ T-cells were cocultured in a 96-well polystyrene plate (U-Bottom, FALCON) and incubated for 4 h at 37°C and 5% CO_2_ (Figure S1 in Supplementary Material). In the target cell death assay, NK cells and CD4+ T-cells were cocultured at an effector:target (E:T) ratio of 10:1. As a negative control, CD4+ T-cells alone were cultured in medium. As a positive control, NK cells were cocultured with the NK cell-sensitive K562 cells at an E:T ratio of 2.5:1. Cell death was measured using 7-AAD staining. In the degranulation assay, NK cells and CD4+ T-cells were cocultured at an E:T ratio of 1:1. As a negative control, NK cells alone were cultured in medium. As a positive control, NK cells were cocultured with the NK cell-sensitive K562 cells at an E:T ratio of 1:1. NK cell degranulation is detected by measuring CD107a expression, a molecule present on the cytolytic vesicles of NK cells. Fluorescently labeled CD107a (2 μl/well) is added at the start of the assay, and monensin (1 μl/well) is added 1 h into the coculture to capture CD107a expression at the cell membrane by blocking the intracellular destruction of the vesicles.

### KIR and HLA Genotyping

Genomic DNA was extracted from PBMCs of healthy donors and HIV patients with the use of the DNeasy^®^ Blood & Tissue Kit (QIAGEN^®^). Using the KIR Typing Kit (Miltenyi Biotec), we determined the presence of five inhibitory KIR genes [KIR2DL1, -2DL2, -2DL3, -2DL5 (A/B), and -3DL1] and six aKIR genes [KIR2DS1,-2DS2, -2DS3, -2DS4 (del/ins), -2DS5, and -3DS1]. Using the KIR HLA Ligand kit (Olerup SSP^®^), we can detect the presence of the KIR3DL1 ligand, HLA-Bw4; as well as the C2 and/or C1 motif on the HLA-C alleles, which are ligands of KIR2DL1 and KIR2DL2/3, respectively. NK cell donors underwent both KIR and HLA genotyping, while CD4+ T-cell donors were genotyped for HLA ligands (Figures S2A,B in Supplementary Material).

Only NK cells that are capable of creating a KIR–HLA bond during maturation will become functionally competent or “licensed” NK cells (28–32). According to its HLA genotype, we determined the licensed and/or unlicensed KIR+ NK cell subpopulations in each NK cell donor. In combination with the HLA genotype of the CD4+ T-cell donor, we determined the possible allogeneic KIR–HLA interactions between licensed iKIR+ NK cells and CD4+ T-cells (Figure S2C in Supplementary Material). Absence of the HLA ligand by the CD4+ T-cell donor resulted in a KIR–HLA mismatch, while the presence of the HLA ligand resulted in a KIR–HLA match. In total, 35 cocultures with one mismatch and 12 cocultures with two mismatches were classified.

### Flow Cytometric Analysis

After isolation, the purity of the isolated NK and CD4+ T-cell suspensions was verified by gating for CD56+/CD16+/CD3− and CD4+/CD3+ cells, respectively. NK cells were also stained for NK cell receptor expression of KIR3DL1, KIR2DL1, KIR2DL2/3, and NKG2A.

Measuring NK cell degranulation (Figure S3 in Supplementary Material), CD56+/CD3− NK cells, and CD3+/CD4+ T-cells were identified in the lymphocyte gate. Subsequently, the frequency of CD107a+ NK cells was determined. In addition, CD107a expression was measured within NK cells that express only one of the four inhibitory receptors (KIR3DL1, KIR2DL1, KIR2DL2/3, and NKG2A). Using Boolean gating, three different single-iKIR+ NK cell populations were designated: KIR3DL1+ (KIR3DL1+/2DL1−/2DL2/3−/NKG2A−), KIR2DL1+ (KIR3DL1−/2DL1+/2DL2/3−/NKG2A−), and KIR2DL2/3+ (KIR3DL1−/2DL1−/2DL2/3+/NKG2A−). The antibodies used in the cocultures are CD107a-BV421 (BD), KIR3DL1-FITC (Miltenyi), KIR2DL1-APC (R&D), KIR2DL2/3-PE-Vio770 (Miltenyi), NKG2A-PE (R&D), CD3-APC-H7 (BD), CD4-PerCP-Cy5.5 (BD), and CD56-BV510 (BD). It should be stated that the KIR2DL2/3-antibody (DX27) shows some cross-reactivity with the KIR2DS2 receptor (33).

To measure the target cell death in the cocultures, lymphocytes were gated using FSC and SSC parameters. Within the lymphocyte gate, NK cells were identified as CD3−/CD56+ cells. To optimize the measurement of death cells (Figure S4 in Supplementary Material), CD4+ T-cells were gated as CD3+/CD4+ cells within the total population instead of the lymphocyte gate. Subsequently, the frequency of 7-AAD+ CD4+ T-cells was measured. The antibodies used were 7-AAD (BD), CD56-PE (BD), CD3-APC (BD), and CD4-BV421 (BD).

### Statistical Analyses

All statistical analyses were done using non-parametric tests. Non-paired analysis was done using the Mann–Whitney *U* test, paired analysis were done by Wilcoxon matched-pairs signed rank test, correlations were analyzed using Spearman’s rank correlation, and differences across more than two populations were analyzed using Kruskal–Wallis tests. Pie chart percentages were based on the median values of each NK cell subpopulation.

## Results

### Demographic Characteristics of the Study Populations

The distribution of men and women among the NK and CD4+ T-cell donors was comparable. The median age of the NK cell donors was 55 years with an interquartile range (IQR) between 43 and 63 years. The median age of the CD4+ T-cell donors was 47 years (IQR: 38–52 years). Within the CD4+ T-cell donors, CD4+ T-cell count (363 cells/mm^3^; IQR: 261.5–490) and viral load (92,700 cps/ml; IQR: 17,900–230,074) at the start of ART, as well as the CD4+ T-cell count (745 cells/mm^3^, IQR: 566.5–877.8) and viral load (20 cps/ml; IQR: 20–35) at the time of sample collection were collected. The duration of the ongoing therapy (2,206 days; IQR: 876–4,767) was included as an additional clinical parameter. These parameters were analyzed for their potential to affect the responses in our *in vitro* cocultures. However, no correlations were found, except for a modest but significant correlation between age of the CD4+ T-cell donors and CD4+ T-cell death (*p* = 0.0053; *R* = 0.2491, data not shown).

### A KIR–HLA Mismatch Between NK and CD4+ T-Cell Donors Resulted in a Significant Increase in CD4+ T-Cell Death and NK Cell Degranulation

**Figure 1:**
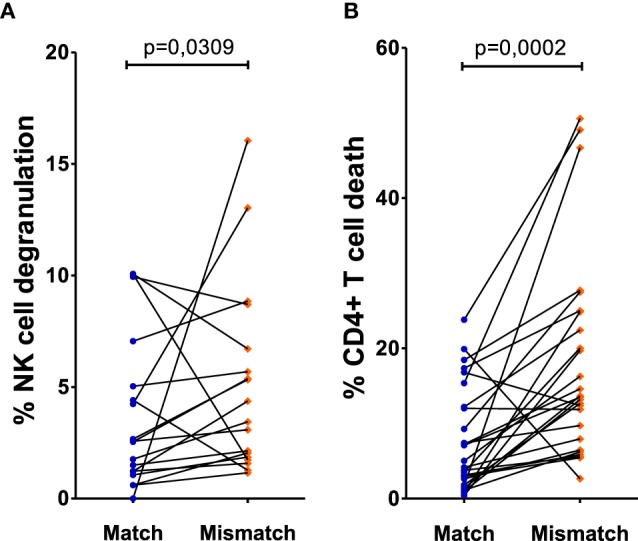
Effect of missing self on CD4+ T cell death and natural killer (NK) cell degranulation: both killer Ig-like receptor (KIR) and human leukocyte antigen (HLA) genotyping of NK cell donors and HLA genotyping of human immunodeficiency virus-1 (HIV) patients resulted in the determination of KIR–HLA mismatches between the NK cells and CD4+ T cells, creating a missing self-context. To exclude NK cell donor variability, we selected the NK cell donors that were put up against CD4+ T cells of two different HIV patients who resulted in a KIR–HLA match on one hand and a KIR–HLA mismatch on the other. In **(A)**, the frequency of degranulating NK cells was compared between KIR–HLA matches and mismatches. Similarly, in **(B)**, the frequency of dead CD4+ T cells was compared between KIR–HLA matches and mismatches. Using a paired (Wilcoxon paired signed ranked test) analysis, a clear increase was seen in a missing self-situation.

To account for NK cell donor variability, we performed a paired analysis by selecting paired cultures where each NK cell donor displayed one or two KIR–HLA mismatches with one CD4+ T-cell donor and no KIR–HLA mismatch with the other (*n* = 22/62 NK cell donors). Cultures with at least one mismatch showed a clear increase in NK cell degranulation, compared to matched cultures with no mismatch (*p* = 0.0309, Figure 1A). CD4+ T-cell death in these cocultures demonstrated significantly more CD4+ T-cell death in a mismatched context compared to matched cultures with no mismatch (*p* = 0.0002) (Figure 1B).

### A KIR–HLA Mismatch as an Initiator for NK Cell Degranulation

**Figure 2:**
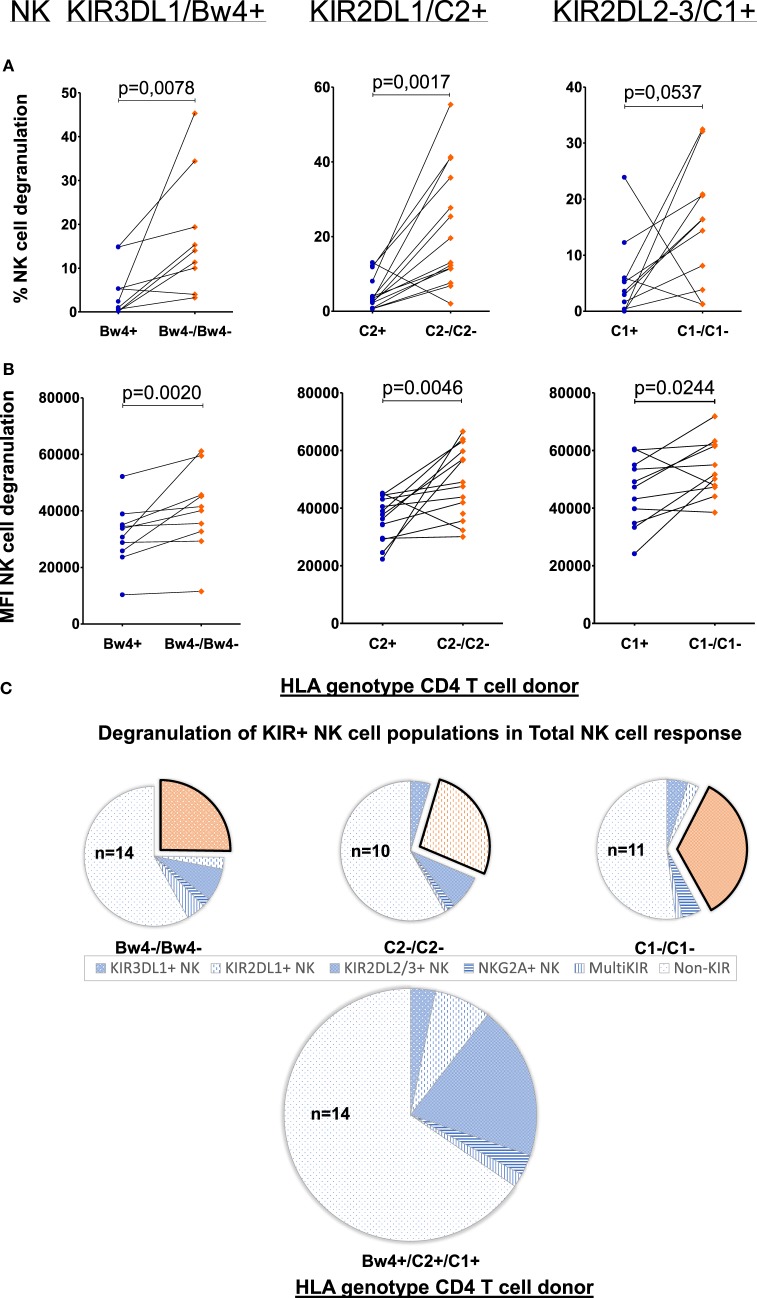
Effect of allogeneic human leukocyte antigen (HLA) genotypes on functionality of single KIR+ natural killer (NK) cell populations: using a Boolean gating strategy, single KIR+ NK cells were isolated to evaluate the impact of the different HLA ligands expressed by the CD4+ T cells on NK cell functionality. A first criteria was the presence of “licensed” NK cells (KIR3DL1/Bw4, KIR2DL1/C2, and KIR2DL2–3/C1, above the graphs). Combination with the HLA genotype of the human immunodeficiency virus-1 (HIV) patients (*x*-axis) resulted in the presence of a killer Ig-like receptor (KIR)–HLA mismatch (orange diamonds) or a KIR–HLA match (blue dots). In graph **(A)**, we compared the frequency of degranulating NK cells by the different single KIR+ NK cells in the context of a KIR–HLA mismatch and match. In graph **(B)**, identically to **(A)**, we compared the intensity of the NK cell response in both the context of a KIR–HLA mismatch and match. Using a paired (Wilcoxon paired signed ranked test) analysis, a clear increase was seen in a missing self-situation. In **(C)**, we show pie charts of the total NK cell degranulation response per mismatched condition, divided into segments of single-iKIR+ and multi-iKIR+ NK cell subpopulations. The single KIR+ NK cell population sensitive to their respective mismatch are highlighted (exploded orange sector). The amount of samples used to calculate median values with the pie chart is constructed is displayed within the pie chart (*n* = x).

We next aimed to investigate whether this apparent allogeneic response was indeed due to specific KIR–HLA mismatches. To this end, a similar paired analysis was performed, this time measuring NK cell degranulation by the NK cells expressing those KIRs to which the ligand was absent in the mismatched coculture. To avoid inhibitory signals received through other KIRs, NK cells expressing multiple KIRs were excluded. Thus, for each NK cell donor, only NK cell subpopulations expressing either KIR3DL1, -2DL1, or -2DL2/3 (single-iKIR) were analyzed. Compared to cocultures where single-iKIR+ NK cells were exposed to HLA-matched CD4+ T-cells, the percentage of degranulating single-iKIR+ NK cells increased under KIR–HLA-mismatched conditions respective to each KIR subtype (KIR3DL1 *p* = 0.0078, KIR2DL1 *p* = 0.0017, and KIR2DL2/3 *p* = 0.0537) (Figure 2A). In parallel, the intensity of degranulation (measured by median fluorescence intensity) increased as well (KIR3DL1 *p* = 0.002, KIR2DL1 *p* = 0.0046, and KIR2DL2/3 *p* = 0.0244) (Figure 2B).

To determine the contribution of the single-iKIR+ NK cells to the general NK cell response, we next differentiated the total NK cell degranulating response into segments of single-iKIR+- and multi-iKIR+ NK cell subpopulations (blue sectors) (Figure 2C). As no additional markers were used, degranulating NK cells not belonging to any of these groups were classified as non-KIR for the purpose of comparison. Each pie chart represents a collection of cocultures where NK cell subpopulations with one of the three KIR–HLA mismatches are highlighted (exploded orange sector). Mismatched conditions were compared to total matched cocultures (*n* = 14). The contribution of single-KIR3DL1+ NK cells to the total NK cell response increased 4.4-fold (21.6/4.93%) in the absence of HLA-Bw4 (*n* = 14), while in the absence of HLA-C2 (*n* = 10), the contribution of single-KIR2DL1+ NK cells to the total NK cell response increased 3.2-fold (26.64/8.4%). The absence of HLA-C1 resulted (*n* = 11) in a 1.4-fold (32.98/24.21%) increase in single-KIR2DL2/3+ NK cells in the total NK cell response.

### The Contribution of an Activating KIR Genotype on NK Cell Cytotoxicity

**Figure 3:**
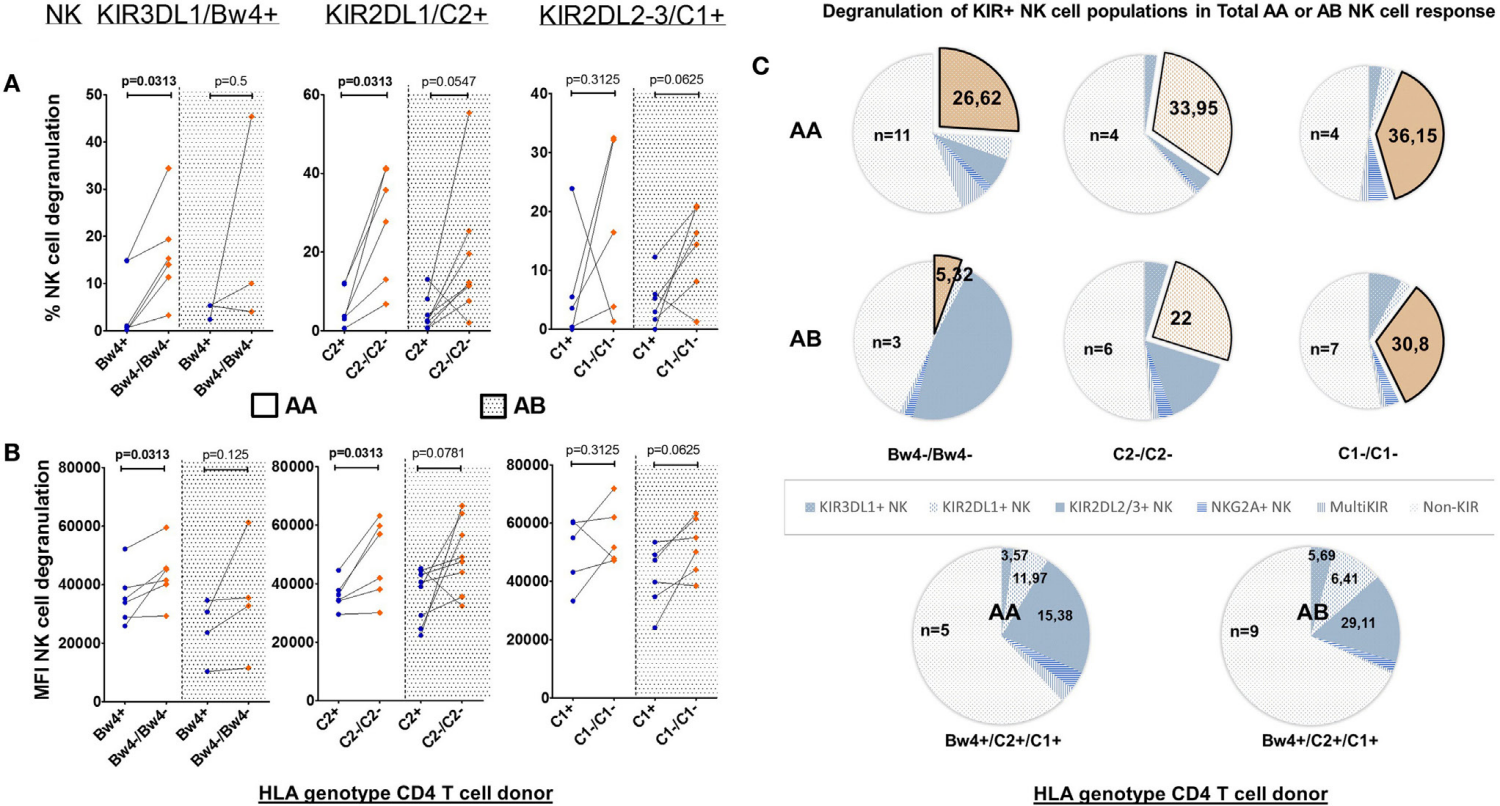
Effect of allogeneic human leukocyte antigen (HLA) genotypes on functionality of single KIR+ natural killer (NK) cell populations in AA and AB NK cell donors: using a Boolean gating strategy, we were able to isolate single KIR+ NK cells to evaluate the impact of the different HLA ligands expressed by the CD4+ T cells on NK cell functionality. A first criteria was the presence of “licensed” NK cells (KIR3DL1/Bw4, KIR2DL1/C2, and KIR2DL2–3/C1, above the graphs). Combination with the HLA genotype of the human immunodeficiency virus-1 (HIV) patients (*x*-axis) resulted in the presence of a killer Ig-like receptor (KIR)–HLA mismatch (orange squares) or a KIR–HLA match (blue dots) with AA or AB NK cell donors. In graph **(A)**, we compared the frequency of degranulating NK cells by the different single KIR+ NK cells in the context of a KIR–HLA mismatch and match. Also AA and AB NK cell donors are segregated by a dotted line. In graph **(B)**, identically to **(A)**, we compared the intensity of the NK cell response in both the context of a KIR–HLA mismatch-match and AA or AB. Using a paired (Wilcoxon paired signed ranked test) analysis, a clear increase was seen in a missing self-situation. In **(C)**, we show pie charts of the total AA or AB NK cell degranulation response divided into segments of single-iKIR+ and multi-iKIR+ NK cell subpopulations in the context of each KIR–HLA mismatch (exploded orange sector) and KIR–HLA match (blue sectors). The amount of samples used to calculate median values with the pie chart is constructed is displayed within the pie chart (*n* = x).

Natural killer cells require at least one activating signal to become activated, independent of their iKIR–HLA compatibility. By dividing, the previously selected NK cell donors based on their aKIR genes (inhibiting A and activating B KIR haplotype, AA and AB NK cell donors), we were able to interpret its influence on the various single-iKIR NK cell populations (Figure 3). Paired tests showed significant differences in a KIR3DL1–HLA–Bw4 and KIR2DL1-C2 mismatch condition, however, only when looking at AA NK cell donors. The frequency (Figure 3A) as well as the intensity of these responses (Figure 3B) was significantly increased (*p* = 0.0313 for all four tests). No differences were seen regarding single-iKIR NK cell degranulation in mismatched conditions between AA and AB NK cell donors.

To measure the impact of the KIR haplotype on the various NK cell populations of the total NK cell response, we again differentiated the total degranulating NK cell population into segments of iKIR+ NK cell subpopulations in the context of each KIR–HLA mismatch (exploded orange sector), this time segregating AA and AB NK cell donors (Figure 3C). In a mismatch condition, the contribution of single-KIR3DL1+ NK cells to the total NK cell response of AA NK cell donors (*n* = 11) increased 7.5-fold (26.62/3.57%) while in AB NK cell donors (*n* = 3) it did not differ (5.32/5.69%). The contribution of single-KIR2DL1+ NK cells in a mismatch condition resulted in a 2.8-fold (33.95/11.97%) and a 3 4-fold increase (22/6.41%) for AA (*n* = 4) and AB (*n* = 6) NK cell donors, respectively. The contribution of single-KIR2DL2/3+ NK cells of AA (*n* = 4) NK cell donors increased by 2.4-fold (36.15/15.38%) in a mismatched condition, while in AB (*n* = 7) NK cell donors it did not differ (30.8/29.11%).

### Comparing the Initial Strengths of the Various KIR–HLA Mismatch Effects

**Figure 4:**
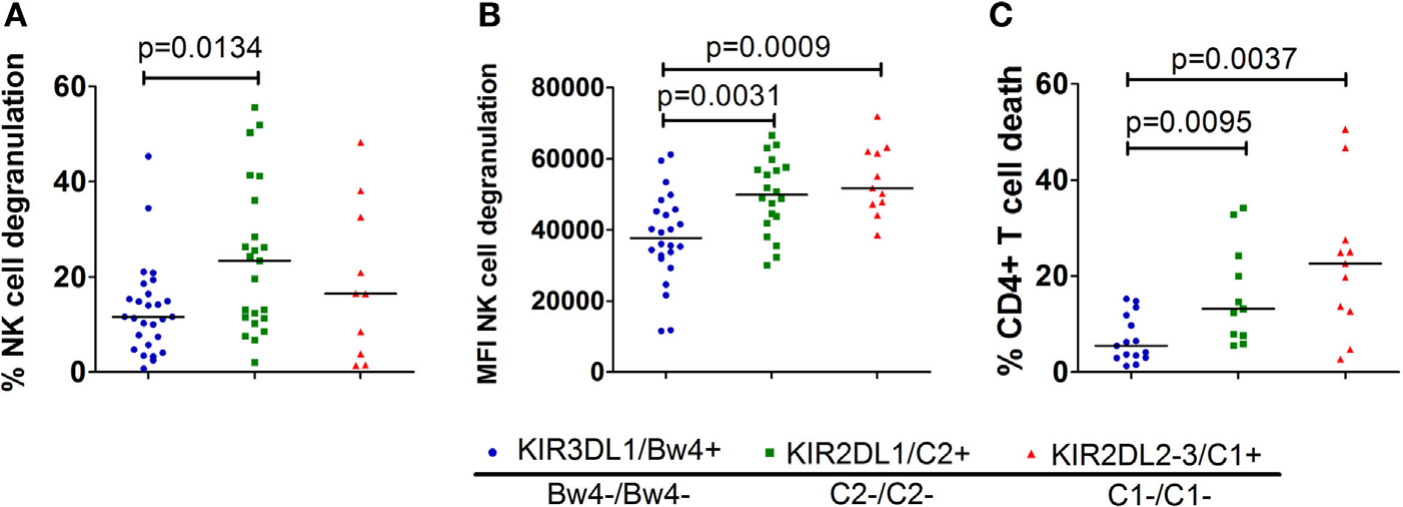
Comparison of the different single KIR+ natural killer (NK) cell populations in the missing self-context: in graph **(A)**, we compared the % degranulating NK cells within each single-iKIR NK cell population in the corresponding missing self-context. Graph **(B)** shows the intensity of the degranulation of NK cells in a missing self-context between the different single KIR+ NK cell populations. In graph **(C)**, we compared the % dead CD4+ T cells in a missing self-context between the different single KIR+ NK cell populations. Using non-parametric analysis (Mann–Whitney *U* test), we demonstrate clear differences between the various single-iKIR+ NK cell populations.

Next, we investigated which single-iKIR+ NK cell population responded the strongest in the absence of their respective ligand. Therefore, we compared the NK cell degranulation and CD4+ T-cell death induced by the different KIR–HLA mismatches (Figure 4). To this end, we selected and compared all cocultures containing one or two KIR–HLA mismatches, regardless of available KIR–HLA matched controls (*n* = 47/124). Significantly more degranulating single-iKIR+ NK cells were seen in the context of a KIR2DL1-C2 mismatch compared to a KIR3DL1-Bw4 (*p* = 0.0134) mismatch (Figure 4A). When looking at the intensity of the NK cell degranulation (Figure 4B), the intensity of the KIR3DL1-Bw4 mismatch response was significantly lower compared to the intensity seen in the context of a KIR2DL1-C2 (*p* = 0.0031) and a KIR2DL2/3-C1 (*p* = 0.0009) mismatch. Because the frequency of dead CD4+ T-cells is a downstream event, it cannot be specifically attributed to a single NK cell subpopulation when multiple KIR–HLA mismatches are present. To compare the impact on CD4+ T-cell death, we chose to select only cocultures with a single KIR–HLA mismatch (*n* = 35/124). Significantly less dead CD4+ T-cells were demonstrated in the context of a KIR3DL1-Bw4 mismatch compared to KIR2DL1-C2 (*p* = 0.0095) and KIR2DL2/3-C1 (*p* = 0.0037) mismatch (Figure 4C). No significant differences were seen between KIR2DL1+ and KIR2DL2/3+ mismatched events in all of the three tests (Figures 4A–C).

### The Predictive Value of a KIR–HLA Mismatch on NK Cell Cytotoxicity

**Figure 5:**
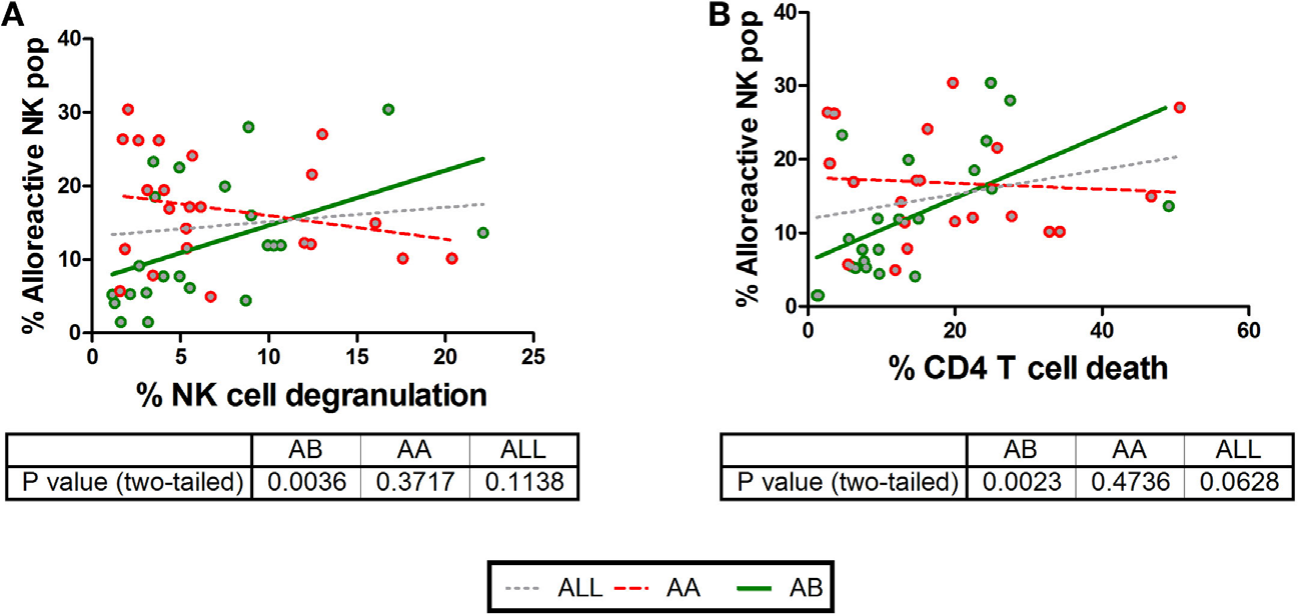
Correlations between CD4+ T cell death and natural killer (NK) cell degranulation with the frequency of NK cells based on killer Ig-like receptor (KIR)–human leukocyte antigen (HLA) interactions and KIR profile. We compared CD4+ T cell death and NK cell degranulation in different KIR–HLA interactions with the presence (green circle) or absence (red circle) of an activating KIR haplotype (AA vs AB). In **(A,B)**, clear correlations were seen with NK cell degranulation (*p* = 0.0036, *R* = 0.594, green line) and CD4+ T cell death (*p* = 0.0023, *R* = 0.616, green line) and in the presence of an activating KIR profile. Correlations were analyzed non-parametrically (Spearman’s correlation). In addition, the correlation irrespective of haplotype is shown as gray dotted line.

Although we clearly show the impact of KIR–HLA mismatches on NK cell responsiveness, it is uncertain to what extent the KIR–HLA mismatch catalyzes the total NK cell response against CD4+ T-cells. Therefore, correlations were calculated between the frequencies of dead CD4+ T-cells or degranulating NK cells (Figure 5) with the pooled frequencies of all single-iKIR+ NK cells subjected to a KIR–HLA mismatch, irrespective of their degranulation state. No correlations between the frequency of the alloreactive NK cell population and CD4+ T-cell death and NK cell degranulation were seen (NK cell degranulation *p* = 0.1667, CD4+ T-cell death *p* = 0.1). Subsequently, similar correlations were conducted segregating AA and AB NK cell donors. Here, the frequency of mismatched NK cells derived from AB NK cell donors did correlate with NK cell degranulation (*p* = 0.0036; *R* = 0.594) (Figure 5A and CD4+ T-cell death (*p* = 0.0023, *R* = 0.616) (Figure 5B).

## Discussion

In this study, we investigated the impact of the different KIR–HLA interactions between NK cells and allogeneic CD4+ T-cells. Inhibitory KIR receptors and their HLA ligands are important inhibiting regulators of NK cell activation. In the absence of their HLA ligand, donor-derived NK cells were capable of eliminating the transformed cells in leukemic patients following haploidentical stem cell transplantation (Haplo-HSCT). NK cells are also reactive to virally infected cells, which are capable of transmitting various viruses during sexual intercourse. In HIV, NK cells have been related to resistance as KIR–HLA incompatibility between sexual partners was previously identified as a factor favoring resistance to HIV (25). Both features suggest a theory wherein a mucosal NK cell response can interrupt the foreign cell-associated viral threat, *via* the HLA incompatibility between partners. To further investigate these theories, we first need to understand the various KIR–HLA interactions with foreign cells, potentially leading to alloreactive NK cell responses. However, such comprehensive knowledge is lacking. Here, we provide a detailed analysis of the intricate balance between NK cell subpopulations when exposed to allogeneic CD4+ T-cells derived from HLA-incompatible HIV patients. NK cell cytotoxicity and CD4+ T-cell death increased in cocultures subjected to KIR–HLA incompatibility. Single-iKIR+ NK cells constituted a significant part of the degranulating NK cell population and were most reactive in a corresponding mismatched condition. Furthermore, of these single-iKIR+ NK populations, KIR2DL1+ NK cells were intrinsically the strongest responders in the absence of their ligand. An activating KIR genotype also contributed to the NK cell cytotoxicity generated by a KIR–HLA mismatch, probably by unleashing the full potential of these NK cells by tipping the balance toward activation.

In accordance with the missing self-theory, we selected only the licensed (and thus functional) KIR+ populations in every NK cell donor to use in the KIR–HLA mismatch analyses (28, 31). We demonstrate a profound degranulating NK cell response in the missing self-context, resulting in increased proportions of dead CD4+ T-cells. To measure the effect of a KIR–HLA mismatch in the purest form, we examined the degranulation of NK cells expressing only one iKIR, as they are unaffected by the presence of unrelated KIR ligands. More single-iKIR+ NK cells degranulated in a missing self-context as compared to its HLA-matched condition. Stronger NK cell responses were seen in the absence of the HLA-C ligand (KIR2DL1–3) compared to the HLA-Bw4 ligand (KIR3DL1). The significant increased proportions of degranulating KIR2DL1+ NK cells within the NK cell population appoint these cells as the strongest intrinsic responders in the missing self-context. While stronger ligand affinity might explain the difference with KIR2DL2/3 NK cells (34, 35), although KIR2DL3 and KIR2DL1 showed similar impact on licensing of NK cells (36). Here, we show that KIR3DL1+ NK cells respond intrinsically weaker in HLA-incompatible conditions compared to KIR2DL+ NK cell responses. Whether these differences are generated by differences between KIR3DL- and KIR2DL-receptors or HLA-B- and HLA-C-ligands is unknown. Interestingly, Jennes et al. (25) associated the genotyped KIR2DL1–HLA-C2 mismatch with protection against HIV, highlighting the cytotoxic potential against HIV transmitting cells of these KIR2DL1+ NK cells in the missing self-context. These cells, if licensed, are also strongly polyfunctional and attracted to the bloodstream during acute primary HIV-1 infection (37). KIR2DL1+ NK cells could therefore play a pivotal role in early mucosal immune responses against cell-associated viruses, especially in the context of HLA incompatibility between partners.

Interestingly, the high allelic diversity of the KIR and HLA genes (38) results in highly variating expression profiles as well as variety in affinity for each other. As the strength of the KIR–HLA bond during NK cell licensing is determined by the affinity and expression levels of KIR and HLA molecules (rheostat model), the magnitude of the missing self-induced NK cell response could highly variate due to these highly polymorphic KIR and HLA alleles (34, 35, 39). Unfortunately, the large repertoire of KIR and HLA alleles and the low amount of specific iKIR–HLA mismatches made it statistically irrelevant to look into allelic diversity in our experiments. However, deep sequencing of KIR and HLA alleles in allogeneic KIR+ NK cell responses against HLA-mismatched, or even matched, CD4+ T cells could explain the high variety in allogeneic NK cell responses in “KIR–HLA similar” conditions.

To further dissect these NK cell responses, we used the genetic KIR data to divide the NK cell donors into inhibitory (AA) and activating (AB) NK cell donors. Although we lacked phenotypical data of the aKIRs, AB NK cell donors were expected to express aKIR receptors on the different iKIR+ NK cell populations (40). Correlations between the size of the mismatched NK cell population and the frequency of dead CD4+ T-cells, and degranulating NK cells were only seen in the presence of an activating KIR haplotype. This shows that KIR+ NK cells in the absence of their ligand still need an activating signal to fully capitalize on the missing self-situation. Unexpectedly, not only the mismatched single-iKIR+ NK cells but also the (matched) KIR2DL2/3+ NK cell contribution to the total NK cell response was increased in AB NK cell donors. Potentially the weak inhibiting signal of the KIR2DL2/3–HLA-C1 interaction, related to weaker affinity (11), is more easily overruled by the activating signals obtained by the aKIRs. Moreover, all but one of these AB NK cell donors contained the KIR2DS2 gene and as the KIR2DL2/3-antibody also stains KIR2DS2 (33), the increase in CD107a+ KIR2DL2/3 in the KIR3DL1− and KIR2DL1 missing self-context might be associated with cross-reactive staining of KIR2DS2 (41). Nonetheless, to fully understand these cell encounters, individual staining of the aKIR+ NK cells is necessary. In addition, Parham et al. (42) demonstrated differences in binding strength between HLA-C and KIR2DL receptor licensing, depending on the KIR (A or B) haplotype, which would majorly impact NK cell cytotoxicity in the missing self-context. However, due to the few KIR–HLA mismatches in the specific AA or AB single KIR+ NK cell populations, the impact of the difference in binding strength is not visible, except for the KIR2DL1 mismatch response in AA individuals (strong KIR2DL1–HLA-C2 bond; *p* = 0.0313).

KIR+ NK cells account for ~40% of the total degranulating NK cell population, and ~80% of these cells express only one single-iKIR, making them highly relevant in KIR–HLA regulated NK cell responses. The impact of a KIR–HLA mismatch is clearly visible with a 4.4-, 3.2-, and 1.4-fold increase of single-KIR3DL1+, -2DL1+, and -2DL2/3+ NK cells, respectively, in the total degranulating NK cell response. In AB NK cell donors, however, only the KIR2DL1+ NK cell population increased in a mismatch context. Again, this highlights the strong intrinsic response of this subpopulation. The contribution of the non-KIR population in matched AB NK cell donors is one-third lower compared to the matched AA NK cell donors (AA = 57.12%, AB = 41.68%). Therefore, the contribution of these mismatches is likely overshadowed by the impact of the activating haplotype on the other matched KIR+ NK cell populations. However, after segregating the donors into AA and AB single-iKIR+ NK cell populations, to few numbers are present to make clear statements or even interpretations. A few drawbacks in this study should be considered. The use of CD4+ T-cells derived from HIV patients creates a more relevant cell population concerning HIV transmission but is accompanied by a low amount of cells retrievable from HIV patients, leading to preferential selection of chronic HIV patients who had been receiving ART for a longer period of time. ART decreases the activity of HIV in the blood, lowering the amount of infected cells and the expression of activating signals. The selected HIV patients did have decent CD4+ T-cell counts, but also low viral loads. As an extended proof-of-concept, however, we primarily investigated the influence of a KIR–HLA mismatch rather than the influence of HIV. Therefore, we used KIR–HLA matched cocultures as control group instead of cocultures with healthy controls. In addition, lack of commercially available flow cytometric labels for the activating KIRs limited analysis of the specific impact of aKIRs on NK cells. However, genetic haplotype segregation allowed for a basic distinction between KIR-profiles. Nonetheless, this still resulted in a limited sample size of certain single-iKIR mismatches, thus limiting the conclusions we can draw from them. We focused on NK cell degranulation, the single cytolytic parameter of KIR+ CD56dim NK cells, to measure the direct cell-associated cytolytic impact of the KIR–HLA mismatch toward CD4+ T cells. Including the measurement of cyto- and chemokines would paint the complete picture on the impact of the KIR–HLA mismatch on NK cell functionality. However, these NK cell functions would rather indirectly impact the cytotoxicity against CD4+ T cells and were therefore not studied. Licensing of NK cells is not only possible through KIR receptors but also through the inhibitory NKG2A receptor, in combination with its HLA-E ligand (43). To what extend KIR or NKG2A control NK cell licensing was recently attained to be directed by a HLA-B dimorphism (44). Nevertheless, in our study, focusing on KIR–HLA mismatches, we limited to iKIR–HLA licensing. New data, however, describe beneficial HSCT outcome with HLA-E incompatible donors, pointing at NKG2A as potential effector receptor (45).

In conclusion, we describe the allogeneic KIR–HLA interactions between NK cells and foreign CD4+ T-cells, as catalyzed by a missing self-context. We measured a clear increase in NK cell responsivity in the absence of an HLA ligand. The impact of the KIR–HLA mismatch differed between and was dependent on the type of KIR, with KIR2DL1+ NK cells as the strongest intrinsic responders in the absence of their HLA ligand. The size of the NK cell population only expressing one single-iKIR was highly relevant and potentially capable of directing the total NK cell response, depending on the HLA genotype of the CD4+ T-cell donor. However, the size of this population only correlated with CD4+ T-cell death and NK cell degranulation after the selection of NK cell donors with an activating KIR haplotype and in the absence of its HLA ligand. These observations highlight the potential of “alloreactive” NK cell responses targeted at HLA-incompatible CD4+ T-cells. As shown genetically by Jennes et al. in HIV-discordant couples (25), we deliver *in vitro* evidence for the cytotoxic impact of HLA incompatibility in encounters between iKIR+ NK cells and foreign CD4+ T-cells.

## Ethics Statement

This study was carried out in accordance with the recommendations of IRB. The protocol was approved by the IRB and a written informed consent in accordance with the Declaration of Helsinki from the subjects was redundant according to the IRB.

## Author Contributions

This research paper was written by PhD-student JH under approval of WJ, who was closely involved with the design, construction, and analysis of the experiments. OG and LK contributed by supervising during development of the manuscript. AC co-conducted the experiments that were performed.

## Conflict of Interest Statement

The authors declare that the research was conducted in the absence of any commercial or financial relationships that could be construed as a potential conflict of interest.
