# Supplementary material for: Impact of the Variable Killer Ig-Like Receptor–Human Leukocyte Antigen Interactions on Natural Killer Cell Cytotoxicity Toward Foreign CD4 T Cells
Source: Front Immunol. 2018 Jul 9;9:1588. doi: 10.3389/fimmu.2018.01588 (PMC6046604; doi:10.3389/fimmu.2018.01588)
Supplement: Supplementary file 1 [file data_sheet_1.docx]

**Supplementary figures**

**Supplementary figure 1**


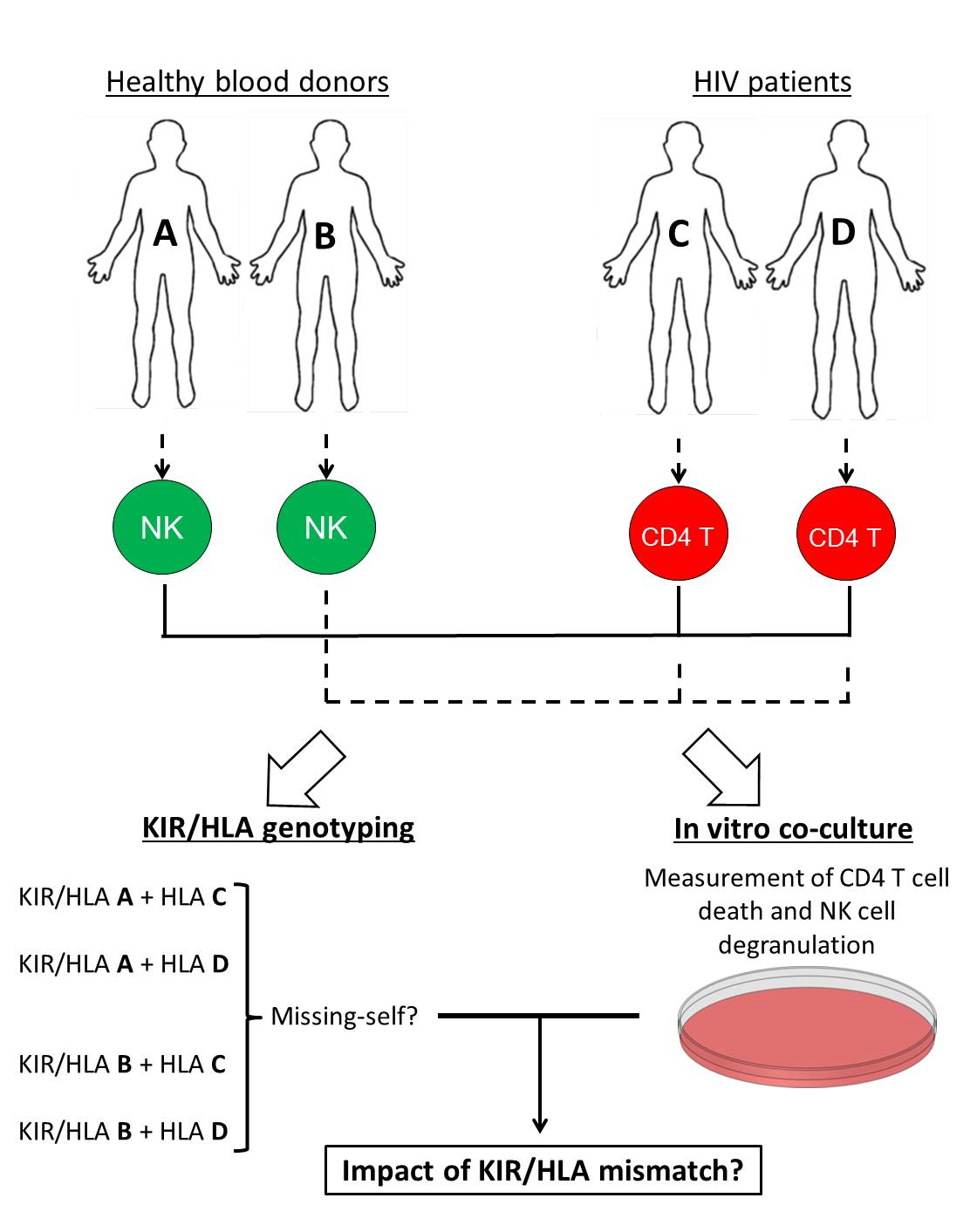


**Supplementary figure 2**

**
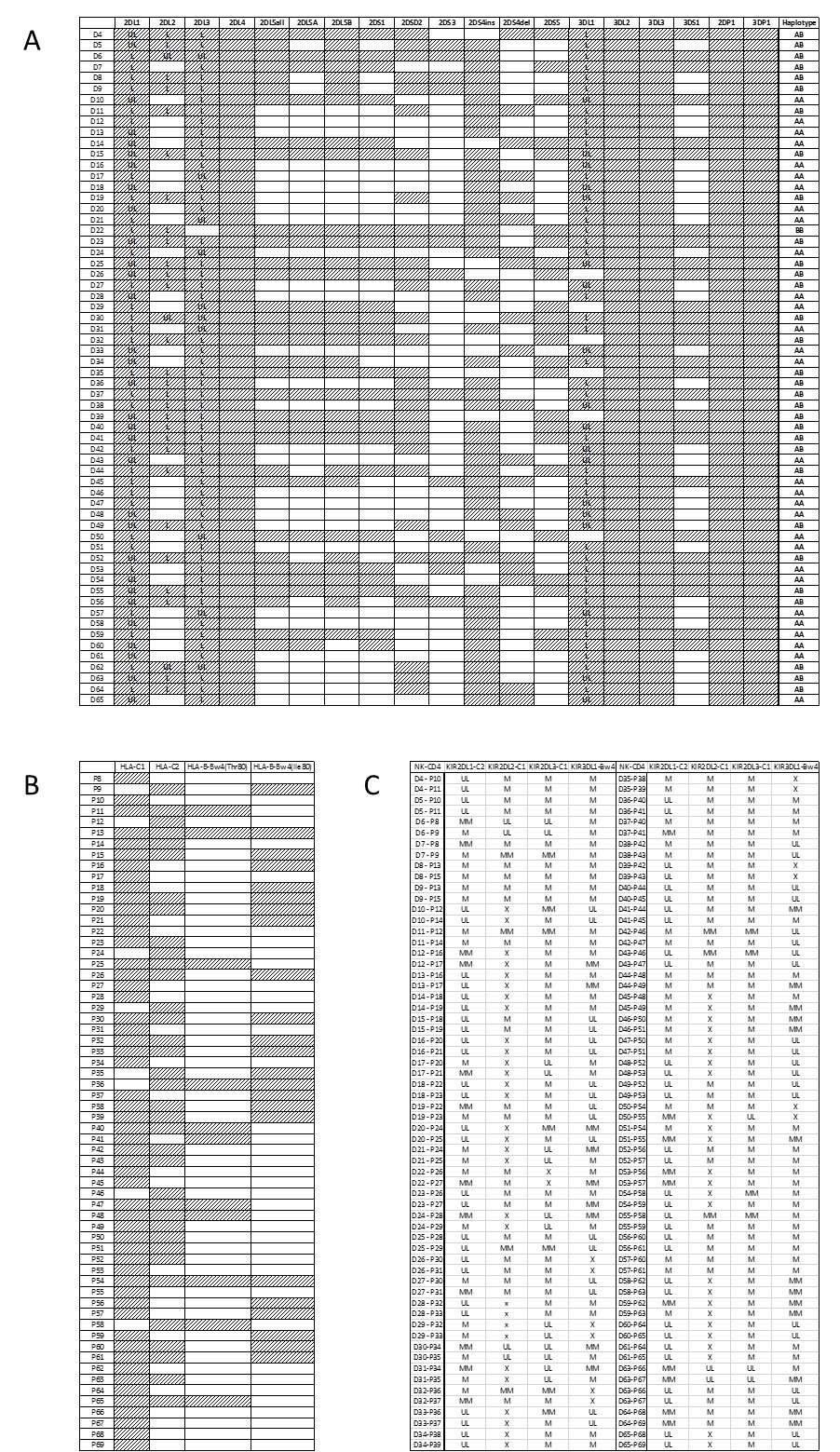
**

**Supplementary figure 3**


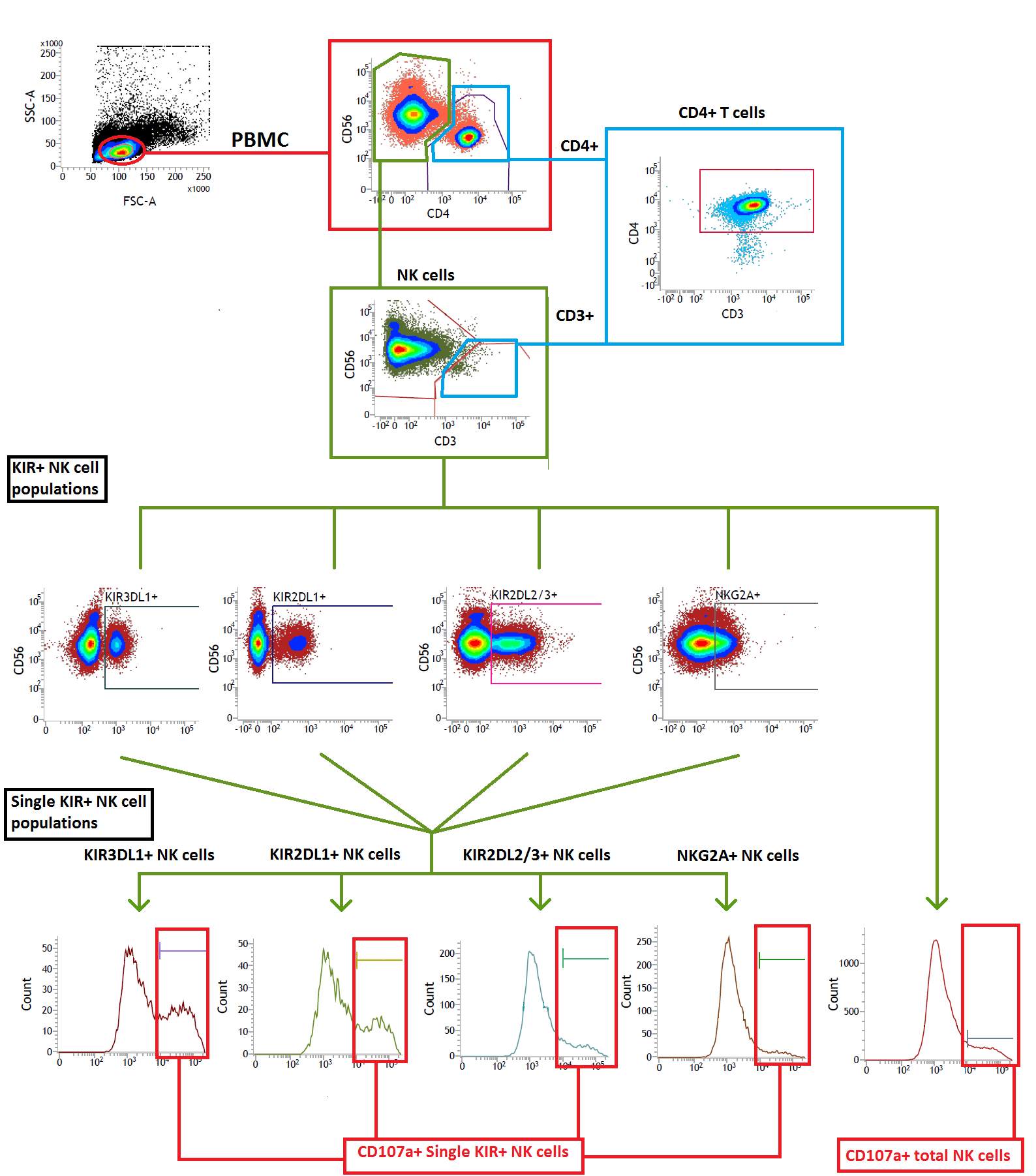


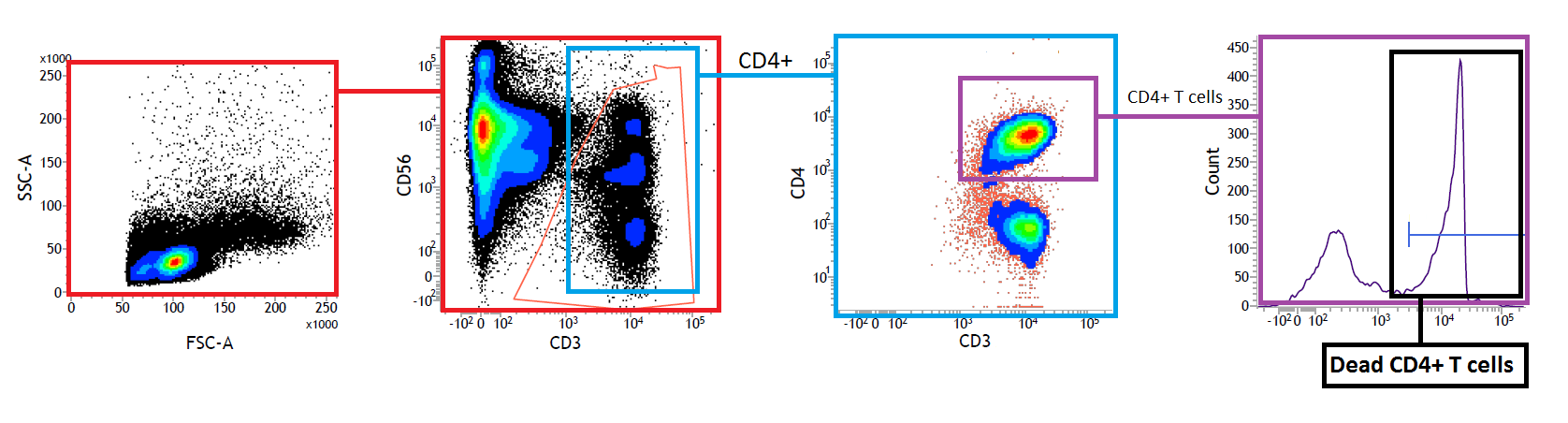


**Supplementary figure legends**

**Supplementary figure 1: Experimental set-up of the genetic and functional experiments:** In every experiment, we isolated the NK cells from two healthy individuals and the CD4+ T cells of two HIV patients. Each NK cell donor was linked with the two HIV patient, resulting in four co-cultures, to optimize the variety in KIR-HLA interactions, resulting in KIR-HLA (mis)matches with the same NK cell donor. Afterwards, the CD4+ T cell death and NK cell degranulation measured in the co-cultures was ascribed to a KIR-HLA (mis)match and was further used for analysis to determine the impact of these KIR-HLA interactions.

**Supplementary figure 2. Tables of KIR and HLA genotyping:** In table A) we show the results from the commercial KIR genotyping kits on DNA from the NK cell donors (D4-D65, KIR2DL1-KIR3DP1) which resulted in knowing the KIR haplotype (AA, AB or BB). In combination with the results from the commercial HLA genotyping kits on DNA from the NK cell donors (HLA-C1/2,-Bw4I/T80), we identified the iKIRs (3DL1, 2DL1 and 2DL2/3) as licensed (L) or unlicensed (UL). In Table B) we show the results from the commercial HLA genotyping kits on DNA from the HIV patients (HLA-C1/2,-Bw4I/T80). We combined tables A) and B), and generated a table C) showing the allogeneic KIR-HLA mismatches (MM) and matches (M). MM and M were only generated by licensed (L) iKIR genes, otherwise a UL or an X mark an unlicensed or an absence of the iKIR gene, respectively.

**Supplementary figure 3. Template of the flow cytometric analysis of the degranulation of NK cells in the NK-CD4+ T cell co-cultures:** For every parameter, CD4+ T cell death and NK cell degranulation, a different co-culture was used. Here we show the template used to analyze the co-culture composed for NK cell degranulation. Starting from the FSC-SSC graph, based on FSC-SSC and cell density we isolated the PBMCs. Next, expression of CD56 and CD4 on PBMCs was displayed in the following graph and CD56+/CD4- cells and CD56-/CD4+ cells were isolated. The CD56+/CD4- cell population was furtherly purified by excluding the CD3+ cells, isolating the CD56+/CD3- cells or NK cells. The CD56-/CD4+ and CD56-/CD3+ cells were combined to gate the CD4+/CD3+ cells or CD4+ T cells. NK cells were stained for the expression of KIR3DL1,-2DL1,-2DL2/3 and NKG2A. The KIR+/NKG2A+ NK cells were used in a Boolean gating strategy resulting in the identification of the Single KIR+ NK cell populations (**KIR3DL1+**/KIR2DL1-/KIR2DL2-3-/NKG2A-; **KIR2DL1+**/KIR3DL1-/KIR2DL2-3-/NKG2A-; **KIR2DL2-3+**/KIR3DL1-/KIR2DL1-/NKG2A-; **NKG2A+**/KIR3DL1-/KIR2DL1-/KIR2DL2-3-). Within the Single KIR+ NK cell populations, we measured the frequency of degranulating KIR+ NK cells. We also measured the frequency of degranulating NK cells in the total NK cell population.

**Supplementary figure 4. Template of the flow cytometric analysis of the CD4+ T cell death in the NK-CD4+ T cell co-cultures:** For every parameter, CD4+ T cell death and NK cell degranulation, a different co-culture was used. Here we show the template used to analyze the co-culture composed for CD4+ T cell death. Starting from the FSC-SSC graph, we selected all events present, as dead CD4+ T cells do not remain in the general PBMC gating, in which mostly living cells reside. Next, all cells were divided based on the expression of CD56+ and CD4+. Within the CD4+ cell population, we isolated the CD4+ T cells as CD4+/CD3+ cells. Within the CD4+ T cell population, the amount of 7-AAD+ CD4+ T cells was measured and were seen as dead CD4+ T cells.
